# Differential Expression of *Tomato Spotted Wilt Virus*-Derived Viral Small RNAs in Infected Commercial and Experimental Host Plants

**DOI:** 10.1371/journal.pone.0076276

**Published:** 2013-10-15

**Authors:** Neena Mitter, Vikas Koundal, Sarah Williams, Hanu Pappu

**Affiliations:** 1 Queensland Alliance for Agriculture and Food Innovation, the University of Queensland, St. Lucia, Australia; 2 Department of Plant Pathology, Washington State University, Pullman, Washington, United States of America; 3 Institute for Molecular Biology, The University of Queensland, St Lucia, Australia; University of California, Riverside, United States of America

## Abstract

**Background:**

Viral small RNAs (vsiRNAs) in the infected host can be generated from viral double-stranded RNA replicative intermediates, self-complementary regions of the viral genome or from the action of host RNA-dependent RNA polymerases on viral templates. The vsiRNA abundance and profile as well as the endogenous small RNA population can vary between different hosts infected by the same virus influencing viral pathogenicity and host response. There are no reports on the analysis of vsiRNAs of Tomato spotted wilt virus (TSWV), a segmented negative stranded RNA virus in the family *Bunyaviridae*, with two of its gene segments showing ambisense gene arrangement. The virus causes significant economic losses to numerous field and horticultural crops worldwide.

**Principal Findings:**

Tomato spotted wilt virus (TSWV)-specific vsiRNAs were characterized by deep sequencing in virus-infected experimental host *Nicotiana benthamiana* and a commercial, susceptible host tomato. The total small (s) RNA reads in TSWV-infected tomato sample showed relatively equal distribution of 21, 22 and 24 nt, whereas *N. benthamiana* sample was dominated by 24 nt total sRNAs. The number of vsiRNA reads detected in tomato was many a magnitude (~350:1) higher than those found in *N. benthamiana*, however the profile of vsiRNAs in terms of relative abundance 21, 22 and 24 nt class size was similar in both the hosts. Maximum vsiRNA reads were obtained for the M RNA segment of TSWV while the largest L RNA segment had the least number of vsiRNAs in both tomato and *N. benthamiana*. Only the silencing suppressor, NSs, of TSWV recorded higher antisense vsiRNA with respect to the coding frame among all the genes of TSWV.

**Significance:**

Details of the origin, distribution and abundance of TSWV vsiRNAs could be useful in designing efficient targets for exploiting RNA interference for virus resistance. It also has major implications toward our understanding of the differential processing of vsiRNAs in antiviral defense and viral pathogenicity.

## Introduction

Tospoviruses (family *Bunyaviridae*, genus *Tospovirus*), infect a broad range of staple and horticultural crops, impacting agriculture in tropical and subtropical areas globally [1]. Transmitted by thrips (Thysanoptera, Thripidae), annual losses due to tospoviruses are estimated to be over $1 billion worldwide [1]. Members of the *Tospovirus* genus are the only plant-infecting viruses in this family, whereas all other genera in *Bunyaviridae* infect humans or animals. Of more than 25 known tospoviruses, Tomato spotted wilt virus (TSWV) is one of the most widely occurring and economically important [2] with a host range of more than 1000 plant species. Crops that are affected by TSWV include bean, lettuce, peanut (groundnut), pepper, potato, tobacco, tomato and numerous ornamental species [3]. 

**Figure 1:**
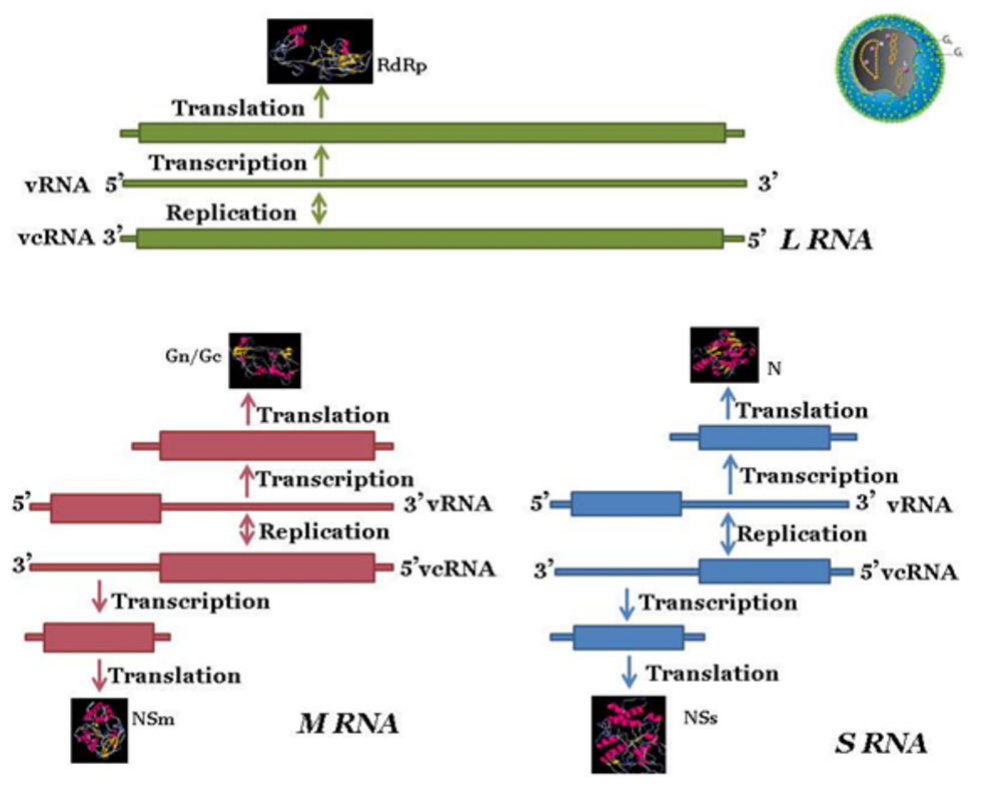
A generalized schematic showing the genome structure and organization of members of genus, *Tospovirus*, using the type member, *Tomato spotted wilt virus* as a model.

TSWV is the best characterized *Tospovirus*. Similar to other members of *Bunyaviridae*, it is an enveloped virus with a tripartite genome composed of three single-stranded RNA (ssRNA) molecules (Figure 1), the large (L), medium (M), and small (S) segments [3,4,5,6,7]. It is distinguished from other animal-infecting members of the *Bunyaviridae* as two (M and S) out of its three genomic segments contain an ambisense gene arrangement [8]. This genome organization allows TSWV to recombine with closely related viruses to generate genome heterogeneity in the face of new challenges [9], and is one of the reasons why tospoviruses are difficult to control or manage. Several TSWV proteins are implicated in infection and transmission of the virus. The NSm protein functions as a movement protein that supports systemic spread of the virus throughout the host [10] and is able to assist the movement of unrelated viruses [11,12]. TSWV glycoproteins are critical for the initiation of vector acquisition by acting as a ligand to trigger entry/attachment into the cell [13,14]. The NSs protein is a silencing suppressor directed against the plant RNA silencing system [15,16].

 RNA Interference (RNAi), a conserved regulatory mechanism in eukaryotes, is triggered by processing of double-stranded (ds) RNA into small RNAs mediated by the interaction of RNAse III Dicer-like proteins ( DCLs), Argonautes (AGO) and RNA-dependent RNA polymerases (RDRs) [17,18,19]. The three main stages of RNAi pathway involves processing of dsRNA by DCLs into small interfering (si) or micro (mi) RNAs, AGO-mediated loading of one strand of these into RNA-induced silencing complex to mediate cleavage of target RNA, and synthesis of dsRNA by RDRs using RNA as a template toward amplification of silencing [20-26]. Plant viruses are strong inducers as well as targets of RNAi, based host surveillance mechanism involved in protection against invading nucleic acids such as viruses, transposons and transgenes [26]. Virus-derived (vsiRNAs) produced as a result of RNAi-based host response to virus infection have been identified in diverse eukaryotic species including plants, fungi, nematodes and *Drosophila* [27,28]. The vsiRNAs are indicative of the activation of RNAi machinery of the host to counteract the viral infection. Virus-specific dsRNA, the trigger molecule for RNAi, in infected cells can be formed by several mechanisms including virus encoded RNA polymerases, base pairing between plus and minus strands of viral RNAs, imperfect folding of self-complementary sequences and action of host encoded RDRs [29]. Three functional RDRs (RDR1. RDR2 and RDR6) have been suggested to play the role of antiviral effectors by using viral RNA as a template to synthesize negative complementary strands [29,30,31]. Analysis of *Arabidopsis* loss-of-function DCL mutants provides genetic evidence that RNA viruses are mainly targeted by DCL4, DCL2 and DCL3 to generate vsiRNAs of 21, 22 and 24 nt respectively [24]. Most vsiRNAs are 21-22 nt in size and are the products of DCL4 and DCL2, which are the most important plant dicers involved in virus-induced gene silencing in *A. thaliana* [32]. Although DCL4 is the major player in vsiRNA production, in its absence, DCL2 is also sufficient to produce 22 nt vsiRNAs, which are biologically active in antiviral silencing. DCL3, which is involved in chromatin modification pathway, targets the viral dsRNA efficiently in the absence of DCL4 and DCL2 [24,32]. DCL1 is a minor contributor to vsiRNA formation in plants infected with RNA viruses [33]. However, siRNAs derived from a DNA virus could be generated by all DCLs [34]. AGO containing effector complexes guide the vsiRNAs to their target molecules [19,35]. Multiple AGO genes might be involved in antiviral defense [36]. The association of siRNAs with a particular AGO protein in plants is primarily dictated by the identity of 5’ nucleotide of the siRNA [37,38].

 The vsiRNAs play a significant role in antiviral defense and host genome modifications and can be the key to our understanding of the viral pathogenicity and host specificity in plants. Cloning and sequencing of plant vsiRNAs suggested that they might be generated from dsRNAs or hairpin regions of ssRNA sequences [27]. Studies using RNA and DNA viruses have shown that vsiRNAs could originate from multiple genomic regions [31,30,39,40]. Development of high-throughput sequencing technologies has allowed the discovery of several vsiRNAs to depict a more accurate scenario about their abundance, complexity and diversity in infected tissues [27]. 

Deep sequencing or next generation sequencing can provide insights into virus-induced plant defense mechanisms and also for characterization of new viruses [41]. Characterization of vsiRNAs by deep sequencing techniques has mostly been done in experimental host plants, however, recent reports have emerged about vsiRNAs in economically important, commercially grown crop species: *Cucumis melo* plants infected with *Melon necrotic spot virus*, *Watermelon mosaic virus* [39] and tomato plants infected with *Tomato yellow leaf curl virus* [42], grapevine plants infected with different viruses [43] and rice plants infected with Rice stripe virus (RSV) [44,45] . Most of the vsiRNA studies are limited to positive-sense RNA viruses except for RSV, a member of the genus *Tenuivirus* with four genomic RNAs and an ambisense coding strategy [44,45]. Xu et al 2012 [45] examined RSV-derived siRNAs in *Oryza sativa* and *Nicotiana benthamiana* through deep sequencing and showed that vsiRNA were more abundant in rice. In case of TSWV, Hagen et al 2011 [46] re-assembled significant portions of the virus sequence from overlapping siRNA sequences and used these to detect a resistance breaking and non-resistance breaking strain of TSWV-infected tomato at time points when the virus could not be detected by conventional means. In this study, we employed the next generation sequencing technology to characterize the vsiRNAs associated with TSWV in two hosts, a commercially important and TSWV susceptible host, tomato (*Solanum lycopersicum*) and an experimental host, (*N. benthamiana*) that is widely used in plant virus research. The results point to significant differences in the vsiRNA abundance of the same isolate of the virus when infecting different hosts. It also shows the differential processing of the three RNA segments of TSWV into vsiRNAs in tomato and *N. benthamiana.*

## Methods

### Plants and Viruses

TSWV was originally isolated from naturally infected peanut (*Arachis hypogaea*) plants and was maintained on experimental host, *N. benthamiana* under controlled conditions in a greenhouse. Tobacco (*N. benthamiana*) and tomato (*S. lycopersicum*, cv. Sunny) plants used in the experiment were grown from seeds under greenhouse conditions at 26°C with 16h day and 8h night. 

### Virus infection of host plants

About three to four weeks post-germination, leaves of tobacco and tomato seedling plants were manually inoculated with TSWV [47]. Virus-infected tissue was homogenized by grinding in 0.01 M sodium phosphate buffer (pH 7.0) containing 0.4% β-Mercaptoethanol and the homogenate was used as the inoculum. Inoculated plants were maintained at 25/18°C (day/night), 14 h days light and observed for development of symptoms. Virus infection of inoculated and systemic symptomatic leaf samples was confirmed at 10, 17 and 20 days post-inoculation (dpi) by ELISA using a commercially available kit for TSWV (Agdia Inc., Elkhart, IN, USA) following manufacturer’s instructions. The infected leaves were also tested by RT-PCR to detect the presence of the genomic RNAs of TSWV using gene-specific primers as described by Bag et al. 2012 [47].

### RNA extraction and analysis

For RNA extraction, systemically infected, non-inoculated symptomatic leaves were collected and snap-cooled in liquid nitrogen and stored at -80°C. Leaf samples were collected at the intervals of 10, 17 and 24 dpi. RNA was extracted from 5g of leaf samples that were confirmed by ELISA as having the same titer in both hosts. Total RNA was extracted using TRIZOL reagent (Invitrogen, USA) following the manufacturer’s instructions. RNA was quantified using Nanodrop and the quality of the total RNA preparation was assessed using 17% denaturing polyacrylamide gel electrophoresis. Total RNA extracted from TSWV-infected tobacco and tomato leaves were used to determine the small RNA profiles by next generation sequencing (BGI Americas Corp., Cambridge, MA).

### Sequencing and Bioinformatics analysis

Small RNA sequencing was performed on the BGI (Americas, Cambridge, MA). Illumina platform with 50 bp reads to get six million clean reads per sample. Adaptor sequence was removed with the Fastx-toolkit package, and reads missing the adaptor were discarded. Trimmed reads were aligned to TSWV (L RNA: NC_002052.1; M RNA: AY744483.1; S RNA: AY744475.1) and the host genome (either *S. lycopersicum* assembly SL2.40 [48], or the v0.42 draft genome of *N. benthamiana* [49] using bowtie, allowing only exact matches [50]. Only reads aligning to a single location on TSWV but not to the host genome were used. BED tools [51] FastQC, HT-seq and custom scripts were used in sequence data analysis. Mountain plots and structure predictions of the TSWV genomic RNA sequence were generated using the RNAfold server [52,53].

## Results and Discussion

**Figure 2:**
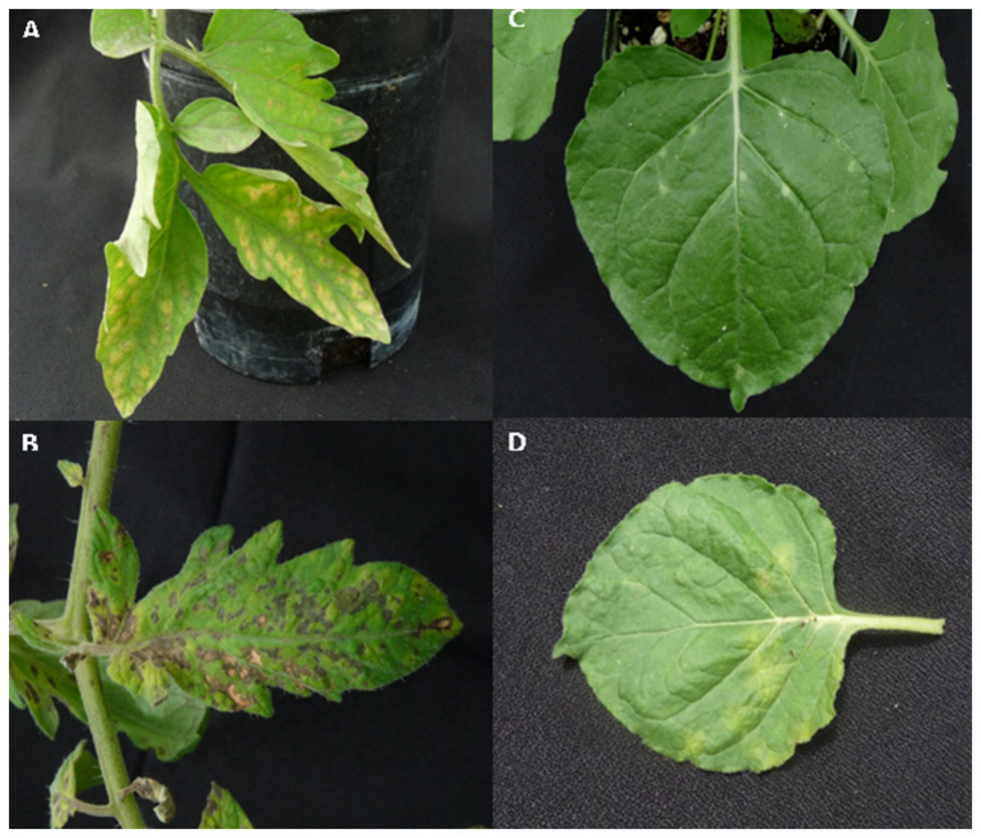
Symptoms of Tomato spotted wilt virus (TSWV) on un-inoculated systemic leaves following mechanical inoculation with TSWV (A) tomato leaf at 17 days post inoculation (dpi) (B) tomato leaf at 20 dpi , (C) *Nicotiana* *benthamiana* leaf at 17 dpi (D) *N*. *benthamiana* leaf at 17 dpi.

*N. benthamiana* and tomato seedlings grown under greenhouse conditions were mechanically inoculated with TSWV. The symptoms caused by viral infection were found to be much more pronounced on tomato as compared to *N. benthamiana* (Figure 2). The infected tomato leaves developed necrotic lesions at 20 days post-inoculation (dpi) whereas *N. benthamiana* leaves showed mild yellow spots. Based on the ELISA data from symptomatic systemic leaves at 10, 17 and 20 dpi, it was decided to use the 17 dpi leaf samples for sequencing with almost same virus titer as represented by OD_600_ value of 1.21 for infected *N. benthamiana* and 1.04 for infected tomato. Total RNA extracted was quantified using Nanodrop and the quality of the RNA was confirmed by polyacrylamide gel electrophoresis. The deep sequencing was done using the Illumina GA2 platform. For small RNA mapping, reads were quality trimmed and adaptor sequence was removed, discarding any reads without adaptor sequence. The trimmed reads were aligned to TSWV and the host genome (either *S. lycopersicum* assembly SL2.40 [48] or the v0.42 draft genome of *N. benthamiana* [49] using bowtie [51]. The sequence has been deposited with NCBI Bio-project database (submission ID: SRP028288, accessible at http://trace.ncbi.nlm.nih.gov/Traces/sra/?study=SRP028288).

**Figure 3:**
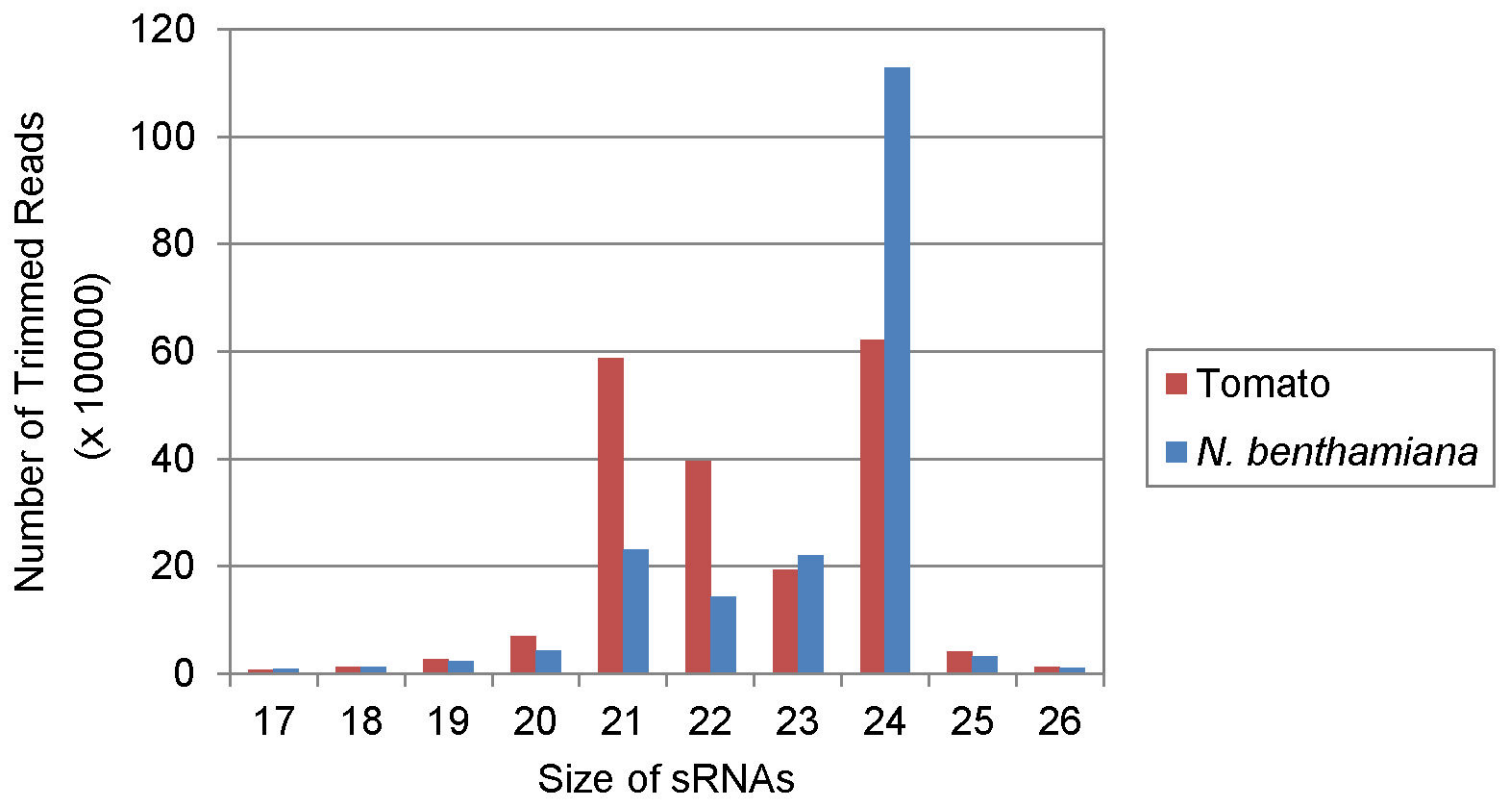
Size distribution of total small RNAs (17-26 nt) in libraries prepared from Tomato spotted wilt virus (TSWV) infected tomato and *Nicotiana* *benthamiana*.

The size distribution of total adaptor trimmed sRNA reads (host + virus) was found to differ in the two hosts. The TSWV-infected tomato sample with 19,940,788 reads had a relatively high proportion of 21 (5,869,267, 29.43%), 22 (3,965,075, 19.81%) as well as 24 nt sRNAs (6,220,116, 31.13%) (Figure 3). The *N. benthamiana* sample with 18,783,645 trimmed reads, however, was dominated by 24 nt (11,291,220, 60.11%) and the 21 and 22 nt sRNAs were only 2,319,252 (12.35%) and 1,439,645 (7.66%), respectively (Figure 3). Difference in predominance of endogenous sRNAs in non- infected hosts with respect to size has previously been reported as 22 nt in *N. benthamiana* [54], 24 nt in *A, thaliana* [55] and 21 nt in *Brassica juncea* [40]. Lin et al [54] compared the total sRNAs of 2-5 million reads in mock and *Bamboo mosaic virus* (BaMV)-inoculated *N. benthamiana* plants. With mock inoculation, the size of siRNAs peaked at 22 nt which represents the endogenous sRNAs. However, after BaMV inoculation, the population of 21nt increased, that of 22 nt decreased whereas there was 30% to 40% increase in 24 nt sRNAs. Because only less than 1% of 24 nt sRNAs were specific to BaMV, most of them therefore, represented the endogenous population. In our studies as well, 24 nt endogenous sRNAs were most dominant in TSWV-infected *N. benthamiana*, whereas the vsiRNAs detected were predominantly 21 and 22 nt in size. Since non-infected *N. benthamiana* was not included in our analysis, we cannot determine whether TSWV infection caused a shift in the size of endogenous siRNAs.

The production of DCL4 dependent 21 nt siRNAs and DCL2 and DCL3 dependent natural endogenous 24 nt siRNAs after virus infection agrees with the previous reports that DCL4 is the major enzyme for generating vsiRNAs against virus infection in plants [24,34,55,56,57] and that DCL2 can substitute for DCL4 in infected plants pointing to the competitiveness of DCL2 [56]. Recently, it has been shown that the level of DCL2 is very high in *N. benthamiana* [58]. This is in contrast to *A. thaliana* in which all four DCLs have fairly similar and relatively low expression levels [24,58]. The size distribution of small RNAs in libraries prepared from RSV infected rice and *N. benthamiana* also showed that 24 nt class was the most dominant accounting for 32.4% in rice and 39.4% respectively [45]. Higher proportion of 24 nt sRNAs compared to 21 and 22 nt have also been reported in cotton plants infected with Cotton leaf roll dwarf virus (CLRDV) [59]. In *B. juncea* the endogenous 21 nt are the most abundant in the non-infected host, however 21 and 22 nt were elevated to a higher level after infection with Turnip mosaic virus [40]. The predominance of 24 nt small RNAs in TSWV infected *N. benthamiana* compared to almost similar proportion of 21, 22 and 24 nt obtained in case of infected tomato (Figure 3) shows that the dynamics of endogenous sRNA generation can vary according to the plant species infected with the same virus implying that the major DCL for producing the endogenous sRNAs is host dependent.

**Figure 4:**
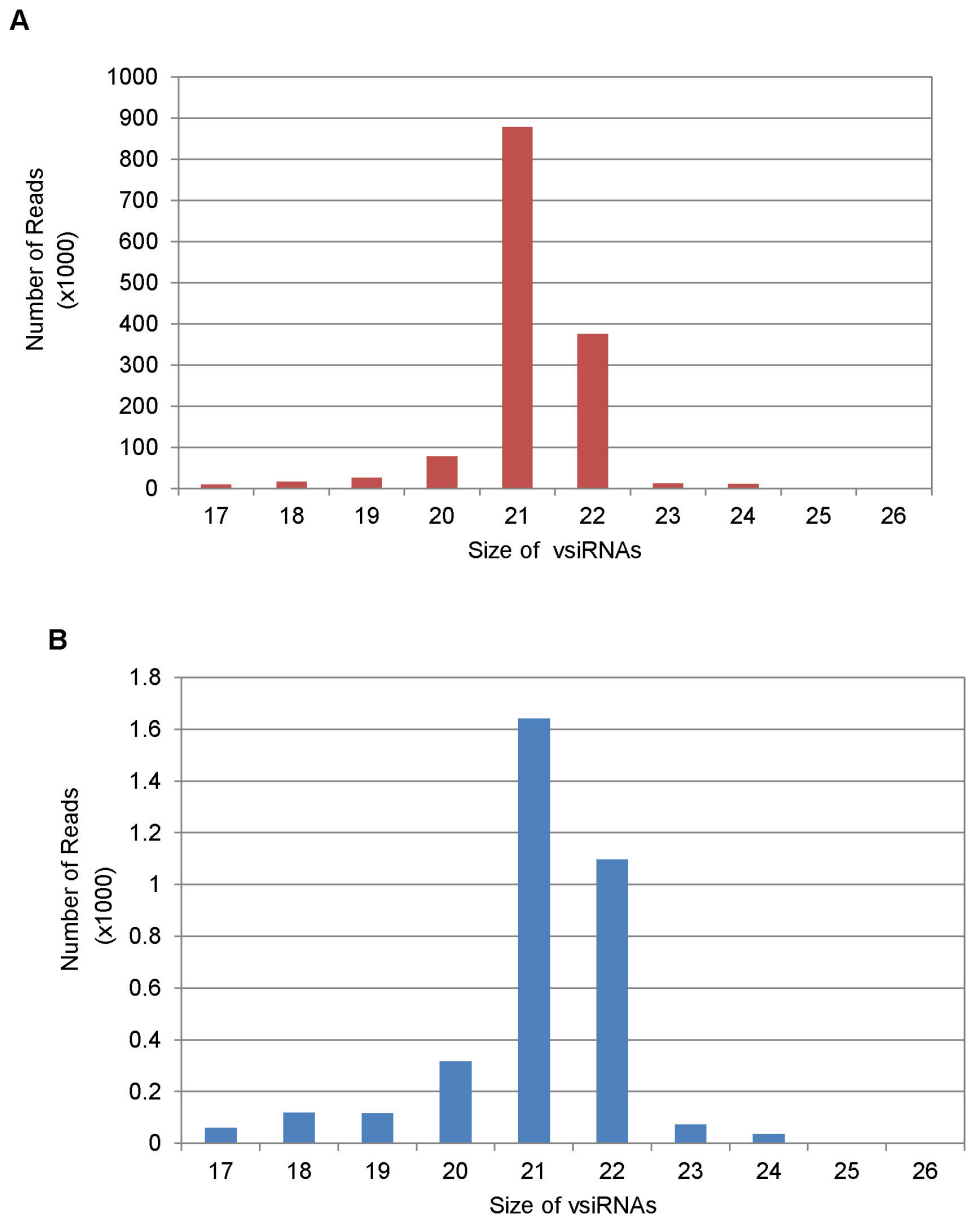
Distribution of vsiRNA sequences matching Tomato spotted wilt virus (TSWV) genome from TSWV infected (A) tomato and (B) *Nicotiana* *benthamiana*. The number of reads obtained for TSWV vsiRNAs (17-26 nt) are shown for both the hosts. Note the difference in Y- axis scale for tomato and *N*. *benthamiana*.

Only those sRNA reads that aligned to a single location on TSWV (L RNA: NC_002052.1; M RNA: AY744483.1; S RNA: AY744475.1) but not to the host genome were used for further analysis. In tomato, 1,412,143 (7.08%) of the 19,940,788 total sequences aligned uniquely to TSWV, but not tomato, while in *N. benthamiana* only 3,433 (0.02%) out of 18,783,645 sequences aligned uniquely to TSWV, but not *N. benthamiana* (Figure 4A and B).Thus, the number of TSWV-specific vsiRNA reads detected in tomato is many a magnitude (7.08%: 0.02% , ~ 350:1) higher then *N. benthamiana* in samples having the same virus titer. This is reflected by the difference in the scale used for the two hosts in Figure 4 A and B and all subsequent figures included in this paper. This shows that different hosts, for the same virus infection, can differ in the population of vsiRNAs. This is probably because of the differences in the efficiency of RNAi machinery to recognize and target the viral genome in a natural versus an experimental host. Lin et al [54] also obtained variation in the percentage of vsiRNAs for BaMV depending on the virus - host system investigated. In *N. benthamiana* plants infected with BaMV, more than 2 million siRNA sequences of 17 to 28 nt, including endogenous and virus derived siRNAs, were obtained of which 3.7% and 17.5% were vsiRNAs in inoculated and systemic leaves, respectively while in *Arabidopsis* only 0.7 to 1.5% vsiRNAs were derived from the same virus. Potato Virus X (PVX) and *Pepper mild mottle virus* (PMMoV) resulted in 15% and 0.3% vsiRNAs in infected *N. benthamiana* leaf samples subjected to high throughput pyro-sequencing [39]. These results show that the vsiRNAs abundance can vary between same virus infecting different hosts or same host infected by different viruses. 

The low number of vsiRNAs reads in infected host plants have also been reported in other host-virus interactions. In RSV-infected rice plants, the unique vsiRNAs reads were only 5,070 out of 917,776 [44]. Silva et al. [59] reported only 51, 607 unique vsiRNAs among the 10,566,377 total reads in cotton plants infected with CLRDV. In tomato plants infected with *Pepino mosaic virus*, more than 90% of siRNAs obtained were of tomato origin with only 1.4 to 3.6% mapping to vsiRNAs in non-enriched libraries from infected plants [60]. Low abundance of vsiRNAs has also been reported in *Watermelon mosaic virus* in *Cucumis melo* and *Tomato yellow leaf curl virus* in tomato [39]. The low levels of TSWV vsiRNAs and the differences observed with respect to vsiRNAs in infected tomato and *N. benthamiana* in the current study and the other reports in literature are probably the result of intrinsic differences in the replication and virus accumulation rates between different viruses and also differences in the efficiency of the RNA silencing machinery to recognize and target each viral genome in the corresponding host. In addition, viral suppressors of silencing also add to the dynamics of levels of vsiRNAs detected in infected hosts. 

Recent genome-level surveys have identified members of the siRNA processing pathways in tomato [61] and *N. benthamiana* [58] and some notable differences unique to *N. benthamiana* were observed. The coding sequence of the *N. benthamiana* RDR1 contains two premature stop codons resulting from a 72 bp insertion with in its RdRP domain [58,62]. Another notable exception reported in both tomato and *N. benthamiana* was in case of DCL3, which appears to have only one dsRNA motif (dsrm) and lacks the DEAD motif in its N terminal helicase region [58]. A tandem pair of dsrms in DCL1, DCL3 and DCL4 and a single dsrm in DCL2 has been reported to be the canonical arrangement in plants [63]. While DCL3 has been shown to generate 24 nt siRNAs from transposon and direct heterochromatin modification, it also produces siRNAs from viruses and their satellite and in concert with DCL2 and DCL4, plays a role in repression of replication [57]. Host-coded RDRs may use viral templates to produce dsRNAs that serve as substrates for the formation of secondary siRNAs [64]. Studies with Tobacco rattle virus (TRV) infection in rdr mutants ( *rdr1, rdr2 and rdr6* ) in *A. thailliana* indicated that in the absence of functional RDRs only a limited amount of viral derived dsRNA was formed , and consequently less substrate was available for DCL- mediated cleavage and vsiRNAs generation despite increasing the amount of viral RNAs [30]. It was further shown that low accumulation of vsiRNAs in *rdr* mutants was not due to lack of TRV replication as the mutants accumulated significantly higher levels of full length viral genomic RNAs and subgenomic RNAs over time. This could also explain the very low levels of TSWV vsiRNAs in infected *N. benthamiana* due to the host’s RDR1 carrying a 72 bp insertion as compared to tomato, while the virus titre was same for both the hosts. *N. benthamiana* is susceptible to a wide range of viruses and is used as a model host in virus-host interaction studies [65]. The low level of vsiRNAs to target the viral genome coupled with higher virus titre supports the hypothesis that the host defence to TSWV is compromised in *N. benthamiana* to a higher level as compared to tomato. It is possible that besides the inactivation of RDR1 by the 72 bp insertion, the loss of dsrm and DEAD motif in its DCL3 may also play a role in its increased susceptibility [58]. There is no premature stop codon in tomato RDR1 and it is also susceptible to 130 different viruses. Thus, it may be a combination of multiple traits involved in weaker anti-viral defence.

As explained above, in addition to inactive RDR1 in *N. benthamiana*, the silencing suppressor, NSs, of TSWV may have further reduced the biogenesis of primary as well as secondary siRNAs in both tomato and *N. benthamiana*. The tospoviral NSs proteins have been shown to have affinity for siRNAs, long dsRNA intermediates as well as microRNA duplexes [68]. Silencing suppression by TSWV NSs resulted in significantly low levels of target siRNAs in agroinfiltrated *N. benthamiana* [66] as compared to the suppression by tombusvirus P19 and *Rice hoja blanca virus* NS3, both known to bind only siRNAs [67,68] implying that TSWV NSs also interferes in the RNA silencing pathway upstream of siRNA synthesis. In addition to siRNAs, it also binds to long dsRNA, in turn preventing them from becoming processed into siRNAs by DCL. Lin et al. [54] reported that BaMV and BSL6 (interfering satellite BaMV) co- inoculated *N. benthamiana* plants accumulated only 0.1% BaMV vsiRNAs, implying that down regulation of BaMV by BSL 6 occurs at a step before the production of siRNAs. It has been shown that the NSs of *Ground nut bud necrosis virus*, a distinct *Tospovirus*, acts as a bifunctional enzyme by removing the 5’ phosphate form dsRNA, the substrate for DCL and having an ATPase activity [69]. It has been observed that DCL does not recognize the dephopshorylated dsRNA [70]. No viral or host encoded partner has yet been identified for *Tospovirus* NSs and there are no reports on differences in functionality of NSs in tomato and *N. benthamiana*. 

In both tomato and *N. benthamiana*, though the number of reads differed significantly for the TSWV specific vsiRNAs (magnitudes of scale difference), the TSWV vsiRNA population was dominated by 21 and 22 nt species in both the hosts (Figure 4). These are considered to be the cleavage products of DCL4 and DCL2. In tomato, the TWSV-specific vsiRNAs constituted 21-22 nt (1252979 reads or 89%), 24 nt (1217 reads, 1%) and others (10%) as shown in Figure 4A. Similar size ratio of 21- 22 nt (2780 reads, 79 %), 24 nt (59 reads, 1%) and others (20%) was obtained in TSWV-infected *N. benthamiana* (Figure 4B). The predominance of 21-22 nt sequences amongst vsiRNAs has previously been reported for several plant viruses and supports the evidence that the 21 nt-long siRNA is the predominant anti-viral silencing component and DCL4 (responsible for the production of 21nt siRNA) is the major producer of viral siRNA [31,34,38,39,40,71]. For all the grapevine viruses tested by Pantaleo et al [44], the prevalent vsiRNAs size was 21 nt corresponding to 65% of total RNA followed by 22 nt species corresponding to 15% of the total vsiRNAs. The RSV vsiRNA population in infected rice plants was also dominated by species of 21 (44.8%) and 22 nt (22.8%) [45]. In case of BaMV-infected *N. benthamiana*, the vsiRNAs were predominantly 21 nt (43~56%) and 22 nt (33~43%) in inoculated as well as systemic leaves with 24 nt vsiRNAs accounting for less than 1% of the total vsiRNAs [54]. The size distribution in all these instances mirrored the pathway of RNAi toward RNA viruses observed in *A. thaliana*, which relies on hierarchical activity of DLC4, DCL2 and DCL3 [24,31,56,57]. The 24 nt vsiRNAs were found to be negligible in both tomato and *N. benthamiana* infected with TSWV (Figure 4). It is not as if the DCL3 pathway is defective in TSWV-infected host plants since we did find a bulk of endogenous 24 nt siRNAs (Figure 3). Donaire et al [30,39] also reported that for most virus-host systems investigated by them, vsiRNAs of 21 nt was clearly the predominant class followed by 22nt vsiRNAs, together accounting for 77.5% of total reads. The exceptions to this are *Cymbidium* ring spot virus (CymRSV)-infected *N. benthamiana*, CLRDV-infected cotton plants and *Tomato yellow leaf curl China* virus*-*infected *N. benthamiana* and *S. lycoperiscum* plants that accumulated 22 nt vsiRNAs to higher levels relative to 21 nt [39,42]. 

**Figure 5:**
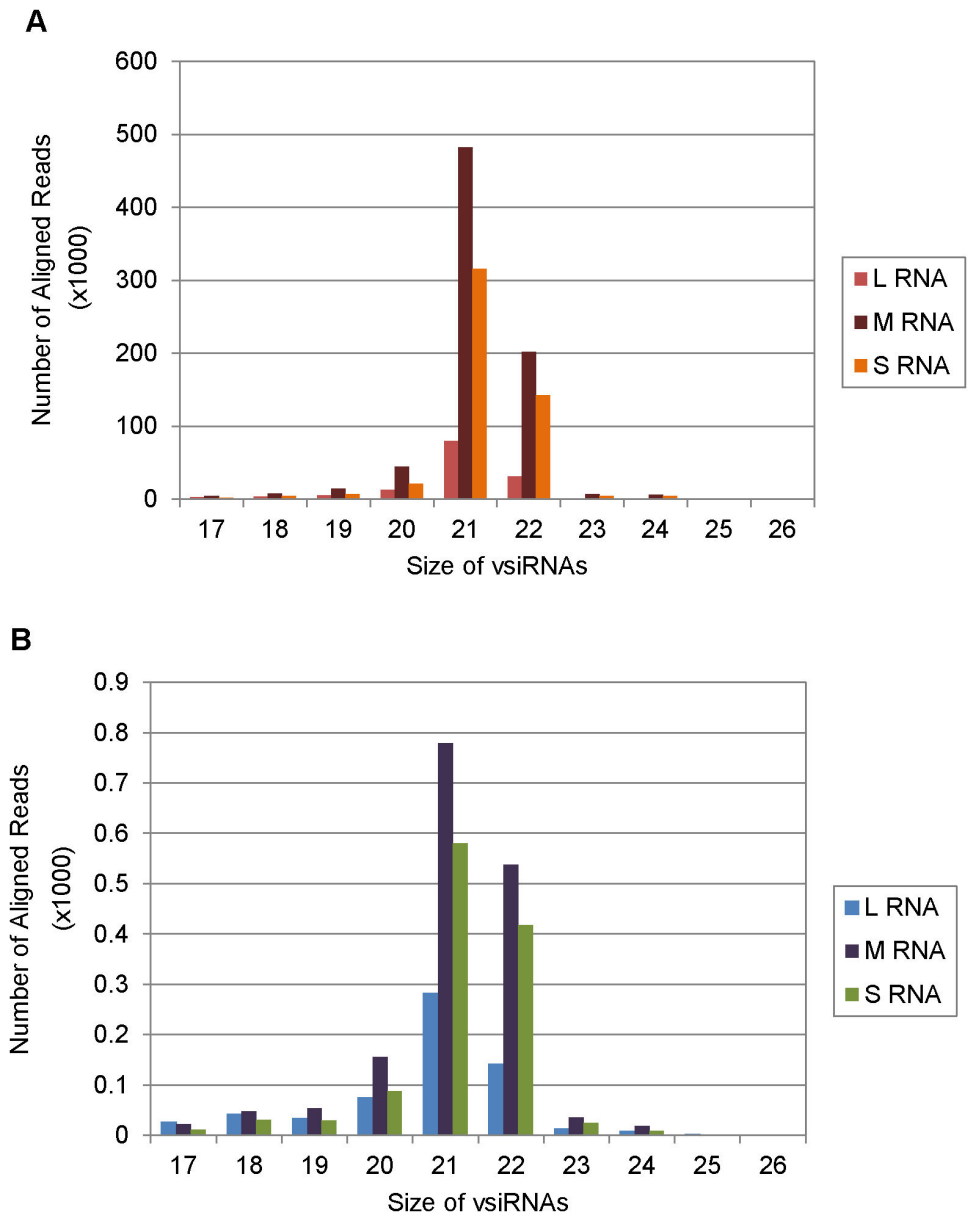
Distribution of vsiRNA sequences matching Large (L), Medium (M) and Small (S) RNA of Tomato spotted wilt virus (TSWV) segments in infected (A) tomato and (B) *Nicotiana* *benthamiana*. The number of reads obtained for TSWV vsiRNAs (17-26 nt) obtained for the three genomic segments are shown for both the hosts. Note the difference in Y- axis scale for tomato and *N*. *benthamiana*.

Differences were observed in the processing of the three TSWV genome segments into vsiRNAs. The large (L) RNA is in negative sense while the middle (M) and small (S) RNAs have an ambisense genome organization (Figure 1). All three RNAs have terminal 8 nucleotides conserved and the various genes are expressed through production of subgenomic RNAs [3]. The L RNA codes for the RNA-dependent RNA polymerase (RdRp). The M RNA encodes a precursor for two glycoproteins (G_N_ and G_C_), and the movement protein (NSm). The S RNA codes for the nucleocapsid protein (N) and the silencing suppressor (NSs) (Figure 1) [4-7]. All three RNAs are tightly bound by the N protein which is further encapsulated by the glycoprotein envelope. The highest number of TSWV vsiRNA reads was detected for M RNA followed by S and L RNA respectively in both tomato and *N. benthamiana* (Table 1, Figure 5). This is in contrast to the expected reads based on segment size. The vsiRNAs in both hosts were predominantly 21-22 nt for all the gene segments. Among the three tospoviral genomic RNAs, the L segment is the largest in size and the number of reads expected was 54% , whereas the number of reads obtained were only 9% and 15 % in tomato and *N. benthamiana*, respectively. This may be due to differences in the relative abundance of TSWV genomic components and also due to other factors making certain regions more suitable for the formation of siRNA. It has been suggested that some special features of genomic areas might affect the substrate affinities for specific DCL activities and thus influence siRNA production [71], and it has been shown experimentally that a hairpin structure of *Cucumber mosaic virus* satellite RNA was targeted by DCL4 with more efficiency [72]. It could also be hypothesized that since the L RNA codes for the RNA-dependent RNA polymerase (RdRp), the enzyme critical for virus replication in infected cell, it is possible that the L RNA evolved in such a way that it is least targeted by the plant RNA silencing machinery. It is interesting to note that in laboratory host *N. benthamiana* the processing of viral RdRp into small RNAs was higher than the natural host tomato. RSV is the only other virus with segmented genome comprising of 4 RNA segments and an ambisense coding strategy for which vsiRNAs have been characterized by deep sequencing in infected rice plants [44,45]. Maximum vsiRNAs reads were obtained for the shortest RNA segment (RNA4), whereas RNA1 (pC1 polymerase), the largest segment, had the least number of vsiRNAs. This is similar to our observations with L segment of TSWV which had the least amount of vsiRNAs in both the hosts tested.

**Table 1:** Tomato spotted wilt virus (TSWV)-specific 21-22 nt virus-specific small interfering (vsi) RNAs reads for Large (L), Medium (M) and Small (S) RNA segments in infected tomato and *Nicotiana benthamiana*.

			**Tomato**		***N. benthamiana***	
**Segment**	**Segment Size (nt)**	**Expected % Reads by Size**	**Actual Number of Reads**	**Actual % Reads Obtained**	**Actual Number of Reads**	**Actual % Reads Obtained**
L	8897	54%	111141	9%	422	15%
M	4767	29%	683891	55%	1316	48%
S	2923	18%	457904	37%	997	36%

The G_N_/G_C_, N and RdRp are organized in the negative sense, whereas NSm and NSs are in the positive orientation of M and S RNAs, respectively (Figure 1). Further analysis of TSWV 21-22 nt vsiRNAs in each gene of the three segments shows that it is the G_N_/G_C_ segment of the M RNA that gets processed to the highest level (35% and 28%) compared to NSm (9% and 8%) in tomato and *N. benthamiana*, respectively (Table 2). Yan et al. [44] obtained a slight bias toward RSV vsiRNAs derived from the complementary strand (53%) in infected rice plants. At first glance it may appear that there was a bias in vsiRNA generation toward genes that are organized in antisense fashion within the TSWV genome in infected tomato as well as *N. benthamiana* (Table 2). However, a closer examination reveals that it is not just a case of bias toward the complementary strand for viral derived siRNAs since a higher percentage (21% and 19%) of siRNAs was derived from NSs (expressed from positive strand) as compared to 10% and 7% N (from complementary strand) in tomato and *N. benthamiana*, respectively. This acquires additional significance since NSs of TSWV has been reported to suppress RNA silencing in plants as well as insects [15,16.73,74]. The results obtained by Schnettler et al [68] showed that all tospoviral NSs proteins analyzed exhibited affinity to small double-stranded RNA molecules, i.e., small interfering RNAs (siRNAs) and micro-RNA (miRNA)/miRNA* duplexes.

**Table 2:** Tomato spotted wilt virus (TSWV)-specific 21-22 nt virus-specific small interfering (vsi) RNAs for genes encoded by Large (L), Medium (M) and Small (S) RNA in infected tomato and *Nicotiana* *benthamiana*.

	**Tomato**		***N. benthamiana***	
**Gene**	**Number of Reads**	**% All Aligned Reads**	**Number of Reads**	**% All Aligned Reads**
G_N_/G_C_	495252	35%	947	28%
Nsm	129143	9%	270	8%
Nc	135703	10%	253	7%
NSs	292781	21%	669	19%
RdRp	105177	7%	392	11%

**Figure 6:**
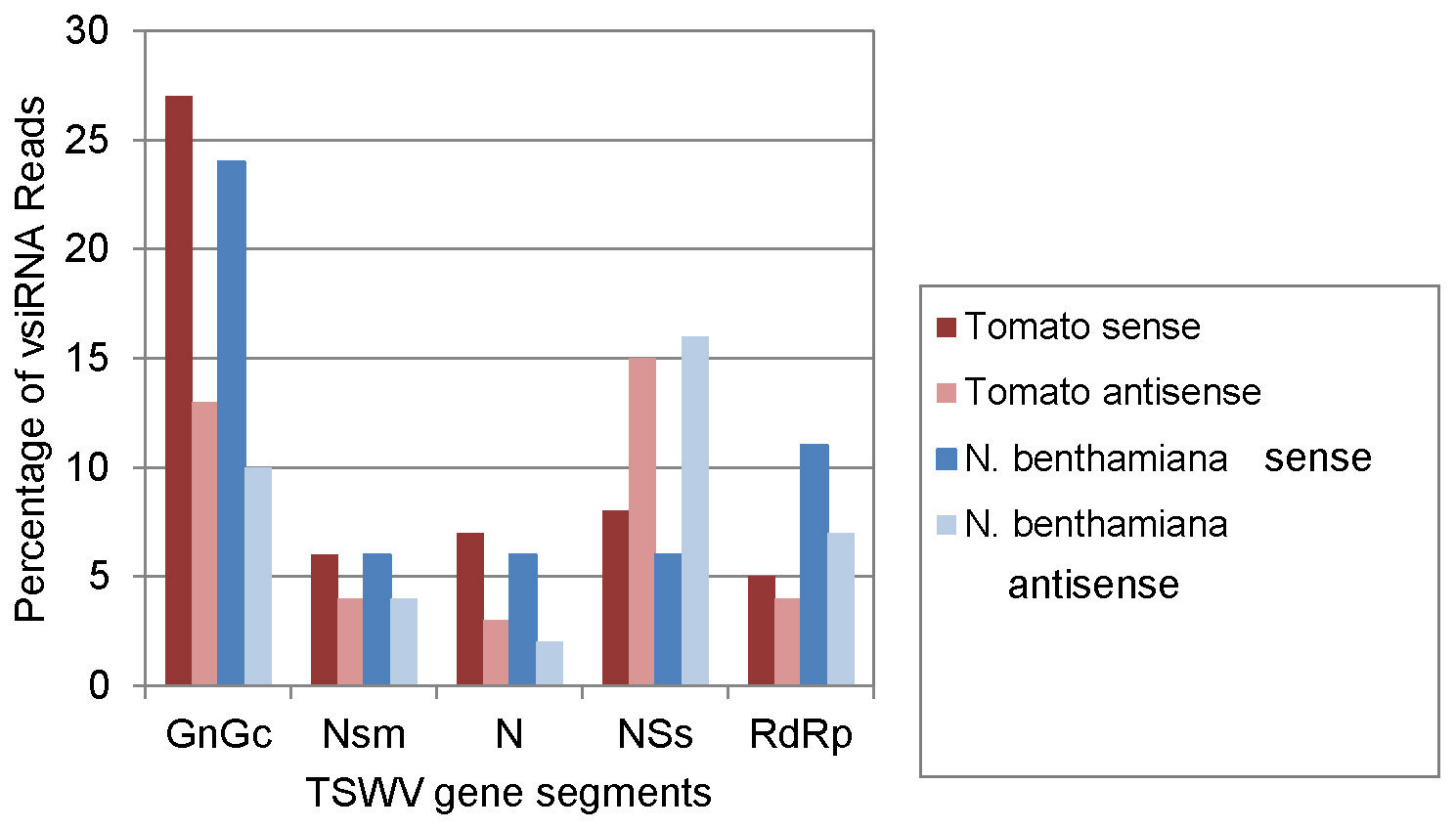
Percentage of Tomato spotted wilt virus (TSWV)-specific antisense and sense vsiRNAs with respect to the coding frame of the genes encoded by Large (L), Medium (M) and Small (S) RNA segments of the virus in TSWV-infected tomato and *Nicotiana* *benthamiana*.

Examination of the relative abundance of sense and antisense viral small RNAs with respect to the coding frame of the different RNA segments of TSWV showed very interesting results (Figure 6). Only in case of the silencing suppressor NSs, the antisense-derived small RNAs were higher than the sense in case of both hosts. For all other fragments (G_N_/G_C_, NSm, N and RdRp) the number of antisense-derived viral small RNAs was less than those of sense siRNAs. 

In CLRDV, a single-stranded positive sense RNA virus containing 6 open reading frames, equivalent amounts of sense and antisense vsRNAs were found in CLRDV infected cotton library [59]. Pantaleo et al [43] reported a clear preference for antisense strands accounting for 75% to 89% of the total vsiRNA reads for three of the grapevine viruses and sense polarity ranging from 61-73% for two of the grapevine viruses in infected grapevine tissue. They did not detect any preferential vsiRNA size class associated with either sense or antisense polarity indicating that the different DCL enzymes do not show strand preference and that sense and antisense polarity might be due to other virus specific factors. Their data suggest that the low abundance of antisense vsiRNAs compared to sense vsiRNAs [38,39,40,75] is not a general feature of plant viruses. 

The preference of viral siRNAs for uredines and adenine and possible selective loading into multiple AGO complexes may provide sequence specificity of RNA target recognition [30,31,40,74,76,77] . It has been suggested that a 5’ terminal U stabilizes vsiRNAs in vivo through association with AGO1, the main AGO protein involved in viral defense [37,38].

**Figure 7:**
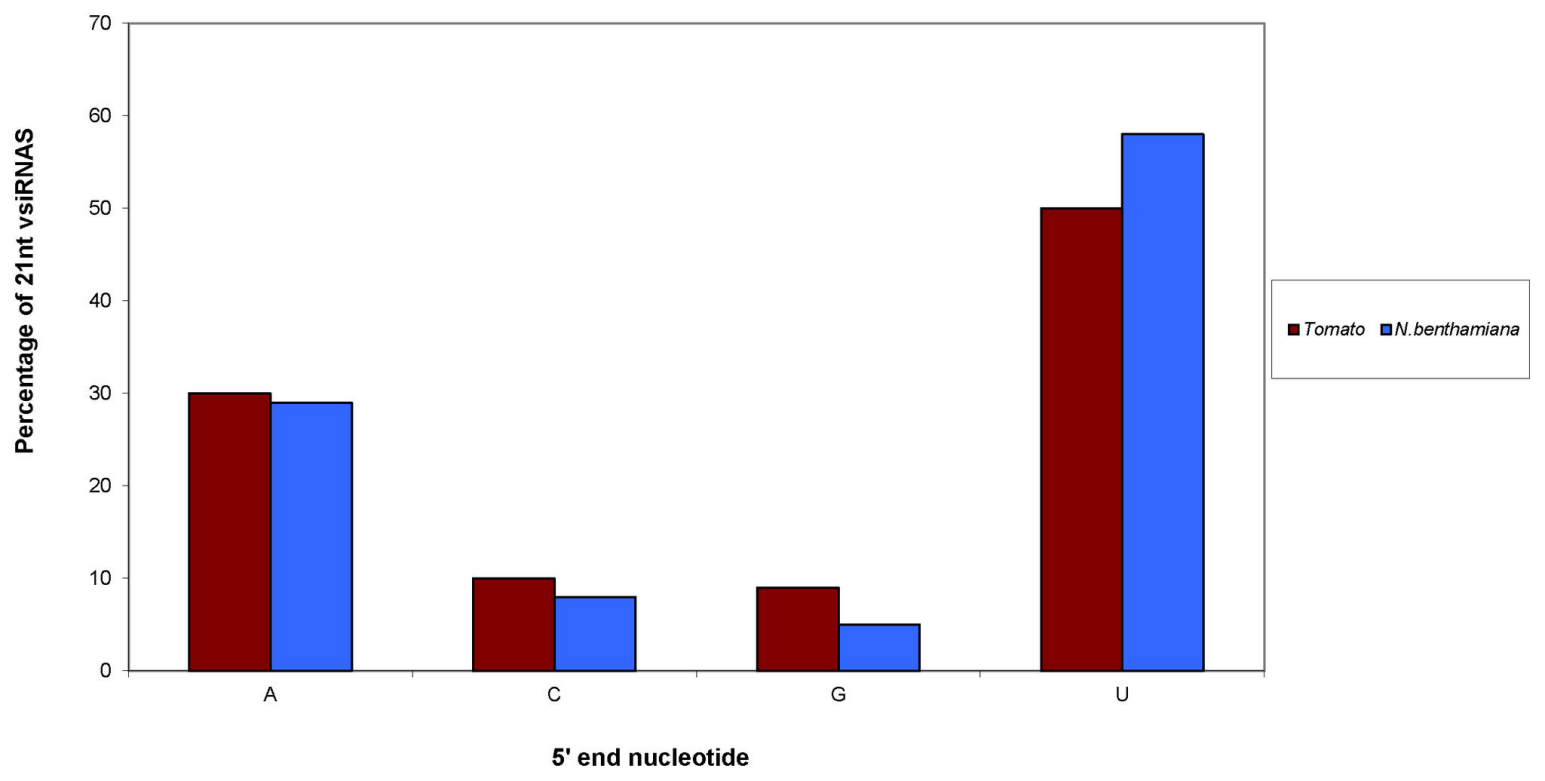
Relative frequency of 5’ terminal nucleotide of Tomato spotted wilt virus (TSWV) vsiRNAs (21 nt) in infected tomato and *N*. ***benthamiana***.

TSWV vsiRNAs in infected tomato and *N. benthamiana* were grouped by the first nucleotide. In both the hosts, the 5’ nucleotide on 21 nt TSWV vsiRNAs was most frequently a Uracil (U) (55%/45% respectively) followed by Adenine (A) at (30%/30%), (Figure 7). Similar results were obtained with analysis of 22 nt vsiRNAs (data not shown). Bioinformatic analysis of RSV-infected rice and *N. benthamiana* plants also revealed a preferential use of U and A residues as compared to Cytosine (C) and Guanine (G) with majority of 21 and 22 nt vsiRNAs showing a strong bias of sequence beginning with a 5’U [44,45]. A similar preferential use of U and A residues compared to C and G in tomato and *N. benthamiana* infected with *Tomato yellow leaf curl china virus* has also been reported [42]. Our observation is in accordance with the previous reporting of AGO proteins with preferred binding affinities for small RNAs having 5′ terminal U (AGO1), A (AGO2 and AGO4), and C (AGO5) [37,38,78]. The low proportion of vsiRNA beginning with a G in our data sets is consistent with the absence of AGO proteins known to prefer siRNAs having a 5′ terminal G [37]. Donaire et al [39] also reported that for most viruses tested, vsiRNAs showed a preference to beginning with U or A and a clear tendency to avoid G.

There was no noticeable difference between the relative frequencies of the 5’ nucleotides in vsiRNAs targeting the different TSWV genes in both the hosts (Figure S1). No differences in 5’ nucleotide frequency were observed between 21 nt and other visrNA subsets, or between those vsiRNAs targeting the sense or antisense strands of genes (data not shown). The GC% of TSWV L (33) M (35.8) and S (34.9) RNA was found to be slightly less than the GC% obtained for vsiRNAs of the respective segments L (36%/36%), M (40%/39%) and S (41%/39%) in both *N. benthamiana*. and tomato respectively. The GC % of (53.8%) of BaMV vsiRNAs was also slightly higher from that of BaMV genome (50.6%) in systemically infected leaves of *N. benthamiana* [55]. DCLs are reported to cut preferentially in GC rich regions and subsequent viral siRNAs have higher GC content than the entire viral genome [30,40,79]. 

**Figure 8:**
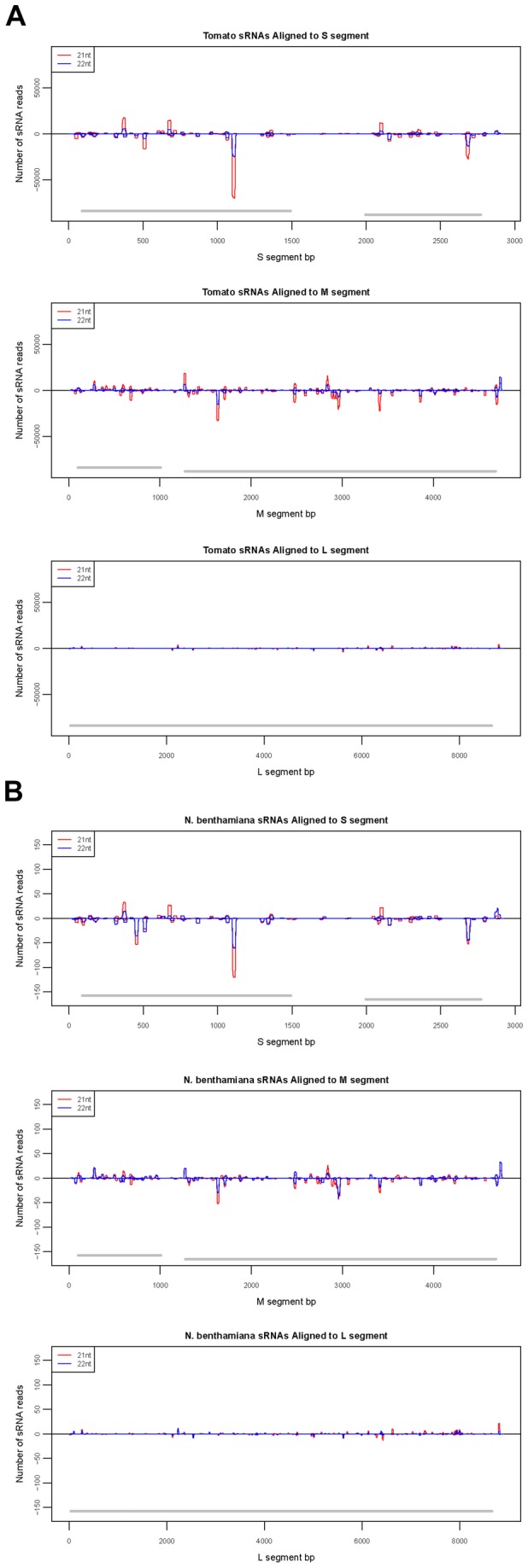
Hotspots for Tomato spotted wilt virus (TSWV) vsiRNAs in Large (L), Medium (M) and Small (S) RNA segments of TSWV in infected (A) tomato and (B) *Nicotiana* *benthamiana*.

Hotspots of vsiRNA accumulation are represented by sharp as well as broad peaks of vsiRNA abundance scattered throughout the viral genome (Figure 8). These peaks are clusters of multiple reads representing several overlapping unique vsiRNA sequences; sharp peaks denote the presence of highly abundant reads with in the cluster. The pattern of hotspots in TSWV-infected tomato and *N. benthamiana* mirrored each other indicating that the host has not made any difference to the processing of vsiRNAs and it seems to be entirely dependent on the virus sequence (Figure 8). Xu et al [45] also reported that similar positions of RSV vsiRNAs hot spots in both infected rice and *N. benthamiana*. Donaire et al [39] reported slightly higher GC content in the hot spots of the viruses investigated compared to other viral genomic regions. 

The hotspot peaks for TSWV vsiRNAs were distributed throughout the gene fragments and no simple relationship was observed between GC distribution and vsiRNAs hot spots. Interesting observation was one particular segment giving a very prominent peak in NSs at positions 1101-1119 in the S segment (starting at 1). This was calculated from positions with over 50000x or 100x 21 nt reads in tomato and *N. benthamiana* samples, respectively. The secondary structures of TSWV RNAs were folded utilizing the RNAfold software as described [52,53]. The Mountain plots obtained with thermodynamic analysis based on minimum free energy (Figure S2) supported the hypothesis that highly structured regions within the viral genome can also serve as substrate for dicer enzymes. Identification of the hotspots of TSWV- siRNA production can be useful for designing efficient targets when exploiting RNAi to silence TSWV. For example, designing a double-stranded structure based on the TSWV M-RNA would appear to be a logical step in attempting to produce RNAi-mediated TSWV resistance in plants. Nevertheless, this aspect of the results needs to be treated with caution, because the next-generation sequencing (NGS) platforms, including Roche 454, Illumina GA, and the ABI SOLiD technologies are strongly biased toward certain sequences. For the short-read platforms (Illumina GA and ABI SOLiD) read-depth coverage decreases with increasing AT content [80]. Previous work in transgenic plants has shown that from all genes of TSWV only the N and NSm gene constructs resulted in resistance, albeit at low frequencies [80]. Subsequent work [81,82] showed that sequences as short as 110 nt from the TSWV N gene were sufficient to efficiently induce RNA silencing. Bucher et al [83] combined N gene sequence fragments (150 nt) of the four major tomato-infecting tospoviruses, TSWV, *Groundnut ringspot virus*, *Tomato chlorotic spot virus*, and *Watermelon silver mottle virus*, in a single small chimeric hairpin (hp) RNA construct and obtained resistance to all four viruses in transgenic plants. 

Our results provide an in-depth investigation and characterization of viral siRNAs from a three component RNA virus with an ambisense coding strategy, and contribute to the understanding of the role of RNAi with respect to the TSWV genome. The number of TSWV-specific 21-22 nt RNA reads detected in tomato was many magnitudes higher than in *N. benthamiana* samples having similar virus titer in both hosts. It is inferred that mRNAs are targeted by corresponding vsiRNAs [44], therefore, it is probable that a lower level of vsiRNAs will result in a higher accumulation of viral transcripts. In case of cassava-infecting begomoviruses, the abundance of vsiRNAs negatively correlated with virus titers in plants undergoing recovery form virus infection, however, in *Cucurbit leaf crumple virus-*infected plants, vsiRNA levels positively correlated with virus titers [84]. Thus, each virus-host combination may be unique in its interaction based on host RNA silencing components and viral genome features. A dynamic equilibrium on feedback loop during viral infection may affect the vsiRNAs levels. Virally coded silencing suppressors add another dimension to this dynamic equation between virus and the host. Our results can be useful in designing antiviral strategies using RNAi against tospoviruses and further understanding of symptom expression and silencing suppression in different hosts. It will be interesting to investigate TSWV infection in various DCL, RDR and AGO mutants with respect to virus replication and vsiRNAs profiles.

## Supporting Information

Figure S1 **The distribution of 5’ terminal nucleotides of Tomato spotted wilt virus (TSWV) vsiRNAs (17-26 nt) derived from TSWV infected tomato and N.** **benthamiana**.
The relative percentage of the 5’terminal nucleotide of vsiRNAs is represented for the individual gene segments of TSWV in both hosts.
(TIF)

Figure S2 **RNAfold analysis of Tomato spotted wilt virus (TSWV) in Large (L), Medium (M) and Small (S) RNA segments derived using the thermodynamic prediction of minimal free energy (MFE) (Lorenz2011), a mountain plot representation of the MFE structure is shown.**
(TIF)
