# Supplementary figures and images for: Differential Expression of Tomato Spotted Wilt Virus-Derived Viral Small RNAs in Infected Commercial and Experimental Host Plants
Source: PLoS One. 2013 Oct 15;8(10):e76276. doi: 10.1371/journal.pone.0076276 (PMC3797105; doi:10.1371/journal.pone.0076276)

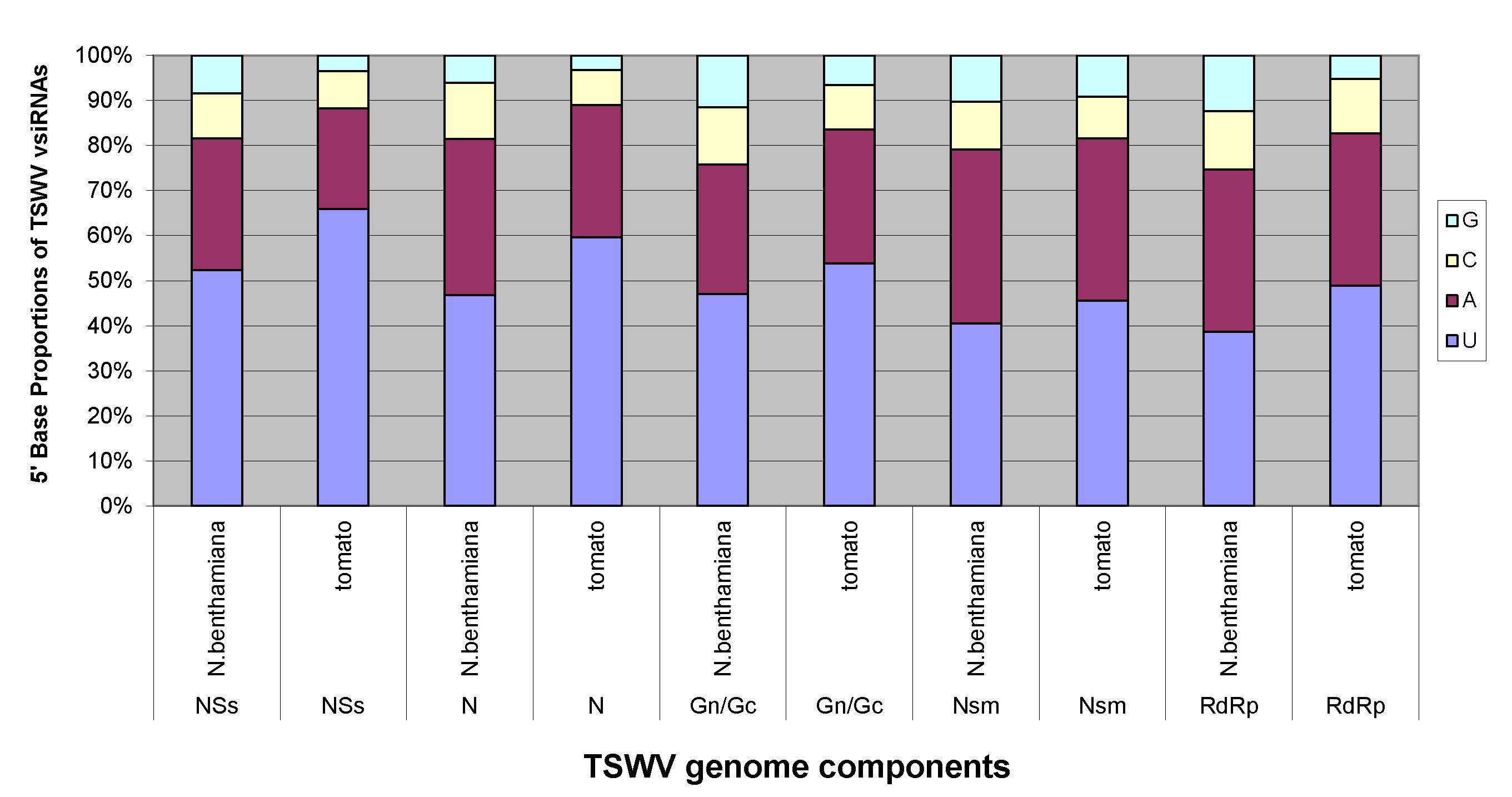

Supplement: Figure S1 — The distribution of 5’ terminal nucleotides of Tomato spotted wilt virus (TSWV) vsiRNAs (17-26 nt) derived from TSWV infected tomato and N. benthamiana. The relative percentage of the 5’terminal nucleotide of vsiRNAs is represented for the individual gene segments of TSWV in both hosts. (TIF) [file pone.0076276.s001.tif]

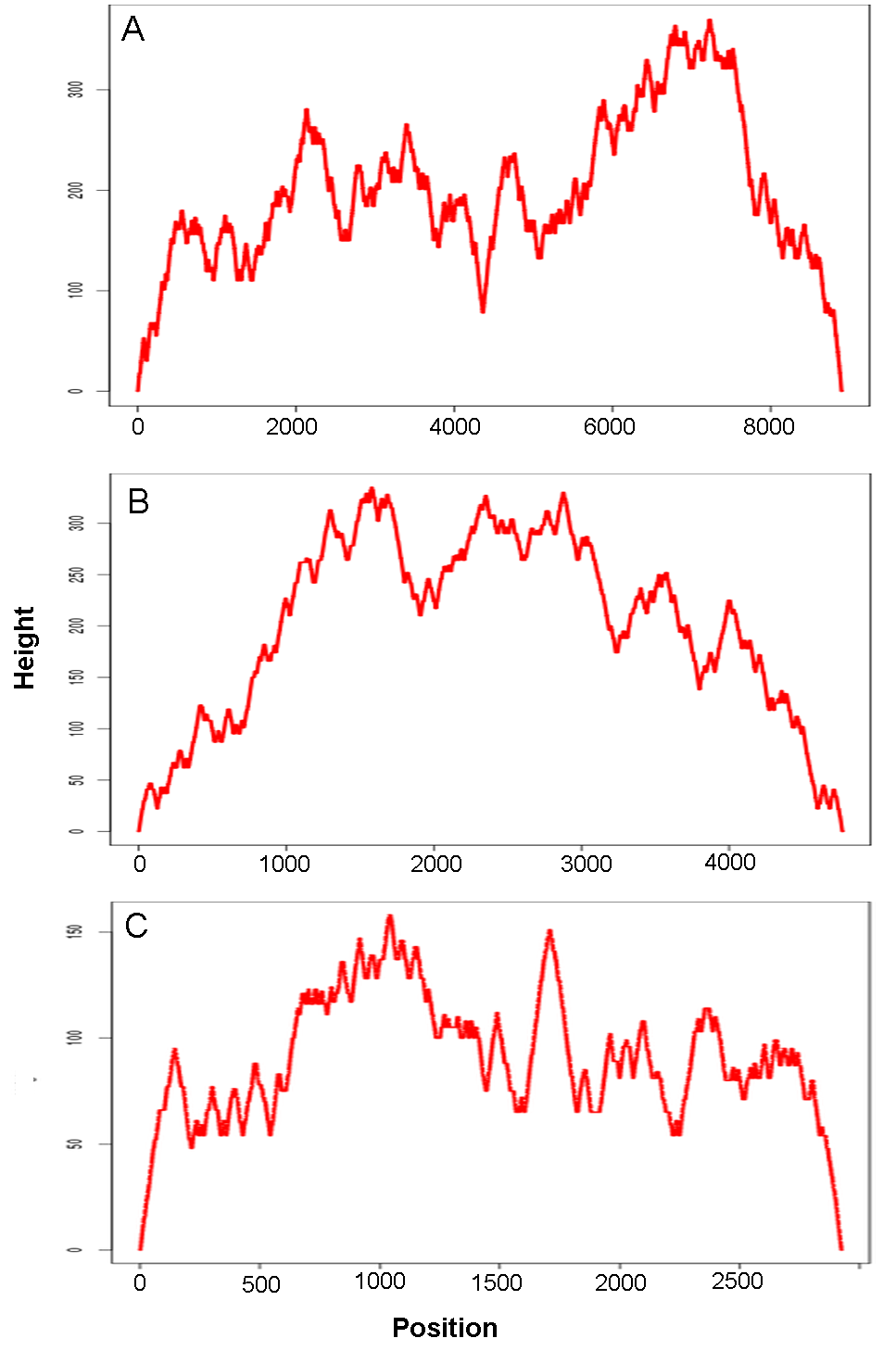

Supplement: Figure S2 — RNAfold analysis of Tomato spotted wilt virus (TSWV) in Large (L), Medium (M) and Small (S) RNA segments derived using the thermodynamic prediction of minimal free energy (MFE) (Lorenz2011), a mountain plot representation of the MFE structure is shown. (TIF) [file pone.0076276.s002.tif]
